# Supplementary material for: Patterns of Circulating Cytokines and Vascular Markers’ Response in the Presence of COVID-19 in Kidney Transplant Recipients Compared with Non-Transplanted Patients
Source: Viruses. 2023 Oct 28;15(11):2166. doi: 10.3390/v15112166 (PMC10675241; doi:10.3390/v15112166)
Supplement: Supplementary file 1 [file viruses-15-02166-s001.zip › Supplementary method 1.pdf]

Supplementary methods

Luminex and Cytokine Cytometric Bead Array (CBA)

After centrifugation, plasma samples were frozen at -80°C and kept until use. The analytes: a disintegrin and metalloproteinase with thrombospondin motifs 13 (ADAMTS-13), growth/differentiation factor 15 (GDF-15), Myoglobin, soluble intercellular adhesion molecule-1 (sICAM-1), myeloperoxidase (MPO), P-selectin, neutrophil gelatinase-associated lipocalin (NGAL), soluble circulating vascular cell adhesion molecule-1 (sVCAM-1), and serum amyloid A (SAA) were measured in plasma by flow cytometry (MAGPIX® Instrument, Luminex Corporation, Austin, TX, USA), using the kit Milliplex Map Human Cardiovascular Disease (CVD) Magnetic Bead Panel 2 - Cardiovascular Disease Multiplex Assay (Temecula, CA, USA). The "cytokines", interleukins (IL) IL-1β, IL-10, IL-2, IL-4, IL-6, IL-8, IL-9, IL-17A, G-CSF (granulocyte colony-stimulating factor), GM-CSF (granulocyte-macrophage colony-stimulating factor), tumor necrosis factor-alpha (TNF-α), interferons-alpha (IFN-α), and -gamma (IFN-γ) were measured using the Cytometric Bead Array (CBA) Flex Set kits and the samples were analyzed in LSRFortessa from BD Biosciences, San Jose, CA, USA. The IL-1β, IL-2, IL-4, IL-17A, and GM-CSF data were excluded from the subsequent analysis due to a high number of zeros, accounting for more than 50% of the data points.
